# The Renin-Angiotensin-Aldosterone System as a Therapeutic Target in Late Injury Caused by Ischemia-Reperfusion

**DOI:** 10.1155/2018/3614303

**Published:** 2018-04-04

**Authors:** Simón Quetzalcóatl Rodríguez-Lara, Leonel García-Benavides, Alejandra Guillermina Miranda-Díaz

**Affiliations:** University of Guadalajara, Institute of Experimental and Clinical Therapeutics, Department of Physiology, University Health Sciences Centre, Guadalajara, JAL, Mexico

## Abstract

Ischemia-reperfusion (I/R) injury is a well-known phenomenon that involves different pathophysiological processes. Connection in diverse systems of survival brings about cellular dysfunction or even apoptosis. One of the survival systems of the cells, to the assault caused by ischemia, is the activation of the renin-angiotensin-aldosterone system (also known as an axis), which is focused on activating diverse signaling pathways to favor adaptation to the decrease in metabolic supports caused by the hypoxia. In trying to adapt to the I/R event, great changes occur that unchain cellular dysfunction with the capacity to lead to cell death, which translates into a poor prognosis due to the progression of dysfunction of the cellular activity. The search for the understanding of the diverse therapeutic alternatives in molecular coupling could favor the prognosis and evolution of patients who are subject to the I/R process.

## 1. Introduction

The ischemia-reperfusion injury (I/R) is a phenomenon that occurs after the restriction of blood supply to the tissues or organs [1]. Abrupt blockage of the blood supply produces an imbalance in the oxygen supply and metabolic nutrients necessary for cell survival at the affected site, which produces a state of hypoxia and blockage of the metabolic processes and the production of energy [2]. However, the reestablishment of blood supply, the increase in oxygen, and the restoration of the metabolic substrates and energy brings about exacerbation of injury in the affected tissue and unchains an exaggerated immunological response that could perpetuate dysfunction of the affected tissue or organ [3].

The renin-angiotensin-aldosterone system (RAAS) is activated locally in the injured cells by the occurrence of I/R which plays an important role in the fate of the injured tissue, as characterized by an increase in stress that the tissue suffers during the assault, and changes caused by I/R lead to changes in the processes of adaptation in the cells subjected to hypoxia [4]. The processes of adaptation involve transformation in the phenotype, function, and structure of the cells involved in the vicinity of the injury [5]. The changes that the cells of the affected tissue undergo will, in the end, cause the deposit of fibrosis and result in another group of cells that are characterized by hypertrophy and dysfunction [6].

The standardization and administration of therapies focused on this system in the late phase of the I/R injury could prevent harmful changes to the affected tissues or organs, improving the prognosis, evolution, and sequelae of the injury process. In this review, we will describe the understanding of the possible mechanisms that unchain activation of the system in I/R injury and the possible therapeutic targets to diminish or prevent sequelae from I/R injury due to the pathological activation of oxidative stress, mitochondrial dysfunction, and autophagy.

## 2. Components of the I/R Injury Connected to the Activation of the RAAS

The I/R injury is an event characterized by multiple physiological components, both early and late [7]. The RAAS plays an important role in the dysfunction of the affected tissues in the late phase of the I/R process. Among the processes involved in I/R injury, some are connected and are perpetuated by the pathological activation of the RAAS, like the formation of reactive oxygen species (ROS) and the reactive nitrogen species (RNS), the disruption of redox signaling, the increase in the concentration of cations in the cytosol, mitochondrial lesion, transcriptional reprogramming, apoptosis, and autophagy [7].

### 2.1. Renin

**Figure 1:**
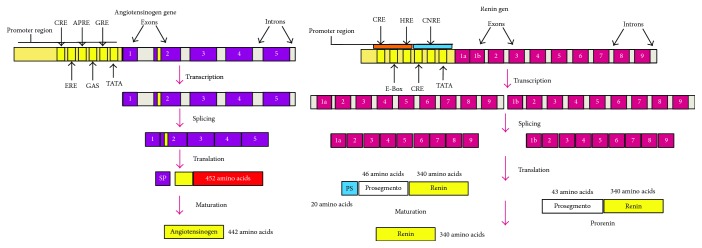
Angiotensin and renin genes.

Renin is an aspartyl protease glycoprotein enzyme that catalyses the restrictive excision of the angiotensinogen (AGT) to angiotensin I (Ang I), an essential component within the processes of the system's activation [8]. The renin gene is found on the chromosome 1q32, contains 9 exons and 8 introns, and encodes different isoforms of the protein by the activation of different promoters and splicing alternatives that are translated into preprorenin [9]. Progenitor cells with the renin secretor phenotype have been described in multiple tissues (cardiac, liver, kidney, nervous, skin, etc.) with the ability to produce renin in case of assault in homeostasis, like changes in perfusion, osmolar changes, inflammation, oxidative stress, and I/R injury. The alterations stimulate cell programming and bring about differentiation and the activation of cells with the renin secretor phenotype [10] (Figure 1).

There are three classic and principal mechanisms of control in the liberation of renin: (a) the activation of glomerular baroreceptors (changes in the diameter of arterioles), (b) the activation of glomerular chemoreceptors or macula densa (changes in the concentration of Na^+^ and Cl^−^), and (c) the activation of *β*_1_-adrenergic receptors (autonomous system and circulating catecholamines) [11]. The acute production of renin in the early phase of I/R injury is realized by the preexisting cells with the renin secretor phenotype and through the quantity of renin stored in the specialized cells located at the site of the injury. In the late phase, plasma concentration is increased by stimulation, transformation, and migration of the groups of cells with the renin phenotype. The number of renin-producing cells is increased, augmenting the renin concentration locally and systemically [5]. Differentiation of the phenotype is mediated primarily by changes in the cellular microenvironment, induced by growth factors like the transforming growth factor beta (TGF*β*) and the epidermal growth factor (EGF), by the activation of survival proteins, hypoxia, stress signals, or unspecific phosphorylation of the membrane receptors like the Ang, tyrosine kinase, and *β*-adrenergic [5]. This kind of transformation of the phenotype is known as epithelial-mesenchymal transition, which offers the differentiated cell the capacity to express genes that in normal physiological conditions are not present, as well as changing its structure and cellular architecture [12]. The process of renin secretion involves, in every case, the activation of adenylate cyclase and the production of cyclic adenosine monophosphate (cAMP) [13]. The renin gene contains a receptor site for cAMP that is crucial for the expression of renin. The production of cAMP is increased by the activation of the AT_1_R receptors, *β*_1_-adrenergic receptors, adenosine, and prostaglandins and the presence of low concentrations of Ca^++^. However, the gene also has other promoters for expression as an element of response to hormones, the nuclear factor kappa B (NF-*κ*B) and signal transducer and activator of transcription (STAT), that also stimulate renin expression, which will be expressed more avidly during the initial and late phase of the I/R injury [11].

### 2.2. Angiotensinogen

**Figure 2:**
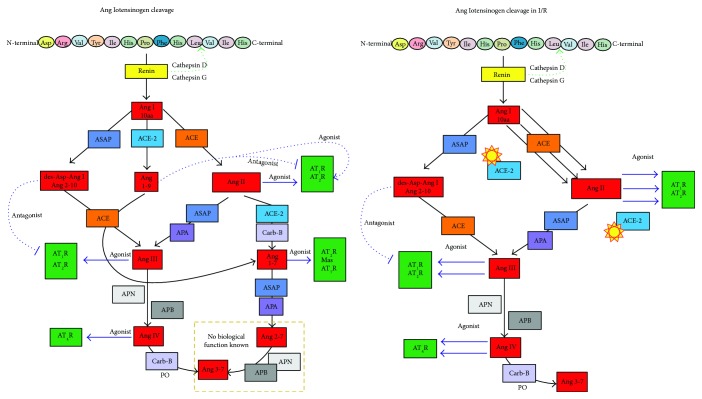
Angiotensinogen cleavage. On the left, the normal process of activation of the RAAS. On the right, the RAAS during I/R; the yellow stars signify blockage of the enzyme and diminished expression.

The AGT is a glycoprotein and serine protease inhibitor that belongs to the superfamily of the inhibitory serpins. The AGT gene is located on the chromosome 1q42-43 and contains 5 exons and 4 introns with more than 12 kb. The AGT has a receptor site for cAMP and is sensitive to activation by the steroid receptor and elements of response to cellular stress [14]. The AGT contains a disulfide bridge between the cysteine 18 and cysteine 138 that gives it a conformational change which permits the access of renin for its maturation [15], and when this bond is oxidized, the affinity of renin for the AGT is augmented, producing an important increase in the concentration of Ang I and Ang II. The AGT in plasma suffers from the processes of glycosylation and polymerization, which confer it a greater affinity for renin [16]. Increments of Ang II are highly influenced by the production of AGT in its polymerized or oxidized state [17] (Figure 2).

Ang I interacts with two enzymes that are similar to each other, the angiotensin-converting enzyme (ACE) and the angiotensin-converting enzyme type 2 (ACE-2) [18, 19]. The ACE produces an octapeptide called Ang II, a larger angiopeptide whose biological function is mediated by the receptors AT_1_R and AT_2_R that are involved in the signaling pathways of survival, cell division, differentiation, the vasopressor effect, hypertrophy, and the modulation of oxidative stress in the I/R process [20, 21]. The ACE-2 produces the nonapeptide called angiotensin 1-9 (Ang 1-9) that has been attributed to antihypertrophic qualities, modulating the signaling pathways of the norepinephrine and IGF-1, probably activated by the bradykinin that potentiates the production of nitric oxide (NO) and arachidonic acid. In addition, it acts as a competitive substrate with Ang I by the ACE, diminishing the production of Ang II on the increasing production of Ang 1-7 [22]. Ang II, when it interacts with the carboxypeptidase B (Carb-B) or the ACE-2, produces Ang 1-7 (Ang 1-7) with the capacity to be antiarrhythmic, a vasodilator of the coronary arteries incrementing the concentration of NO, an antifibrotic to attenuate hypertension, among other functions, through the stimulation of the AT_2_R receptor, the MAS, and the Ang type 7 receptor (AT_7_R). Even, the RAAS system links the mitochondrial pathways in the treatment of I/R-induced injury in cardiac cells because activation of the Ang 1-7/MAS receptor could be a possible adjunct to ischemic preconditioning during cardiac injury induced by I/R [23–25].

#### 2.2.1. Mechanisms of Angiotensinogen Regulation in I/R

The secretion of Ang is regulated locally in the affected tissues on being transformed in the plasma by circulating renin and the production of Ang I in the tissues and later of Ang II [26, 27]. More than 90% of Ang I and 75% of Ang II are produced locally in the injured tissues during the I/R process. Multiple promoters among which regulate the expression of the Ang gene: mineralocorticoids, elements of response to cAMP, steroid hormones, and elements responsible for the acute phase, among others, that are activated during the stages of stress in the cell that are unchained during the I/R process [28, 29]. Meanwhile, the rest of the metabolites are regulated by the activity and concentration of the enzymes responsible for cleavage depending on the state of stress the cell is experiencing, promoting the state of differentiation, survival, transformation, or apoptosis [30, 31].

### 2.3. Aldosterone

**Figure 3:**
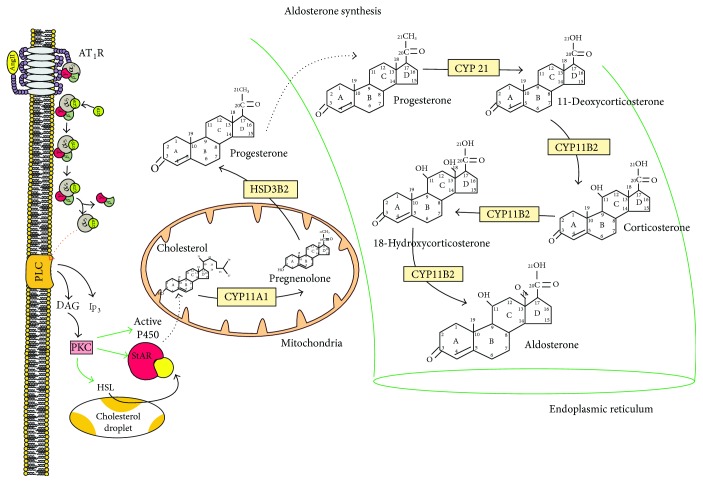
Aldosterone synthesis. The process starts with the activation of the AT_1_R; the G protein is uncoupled and segmented in two fragments. The subunit alpha will activate the phospholipase C which will be produced from PIP_2_, DAG, and IP_3_; the DAG will activate the PKC, and then this will phosphorylate the P450 complex, the hormone-sensitive lipase, and the StAR family of cholesterol transporters, which will lead to the production of aldosterone.

Aldosterone is a steroid hormone and a mineralocorticoid with the most influence in mammals on the control of fluids and electrolytes [32, 33]. The physiological production of aldosterone occurs in the glomerulosa zone of the suprarenal gland; however, it can be produced in other tissues like those of the heart, kidney, blood vessels, and adipocytes [34–36], depending on the state of stress in the cell, where pathologically steroid production is increased. The production of aldosterone involves the activity of multiple enzymes and cholesterol transporters [37, 38] (Figure 3). The cholesterol by endosomes, lysosomes, or peroxisomes is the cholesterol transports to the internal mitochondrial membrane. The main cholesterol transporter is constituted by a family of proteins called steroidogenic acute regulators (StAR), proteins with a high affinity for binding with cholesterol that have capabilities to transport to the mitochondrial membrane, and key proteins in the initiation of steroidogenesis that are phosphorylated during the process of I/R injury [39, 40]. Among the enzymes involved in the liberation, transport, and production of steroids during the I/R phenomenon is the hormone-sensitive lipase that liberates cholesterol from the droplets stored in the cytoplasm to be transported to the mitochondria [41, 42]. The family of cytochromes P450 [43, 44] that are activated by I/R injury, to which the CYP11A1, the 21-hydroxylase (CYP21) and the aldosterone synthase (CYP11B2), the CYP11A1, and the CYP11B2 belong, is found in the internal mitochondrial membrane, while the CYP21 is found in the smooth endoplasmic reticulum (ER). The transporters necessary for the steroidogenesis are active and are highly regulated by the activity of the AT_1_R and the AT_2_R that favor the phosphorylation of the PKC [26]. The transporters have the ability to activate StAR and the enzymes of the cytochrome P450 and the increase in expression of the sterol response element-binding proteins (SREBP) [45]. They are sensitive to esters of cholesterol oxidized during the acute phase of I/R injury, and that maintains the active production of steroids through the activity of AGT metabolites in the late phase of the I/R injury [46, 47]. All of these changes in the expression of enzymes in the cells with I/R injury are known as transcriptional reprogramming [48]. Transcriptional reprogramming can be mediated by the ROS and RNS that, together with the metabolic changes and the signaling pathways mediated by the toll-like receptors (TLRs), activate the signaling pathways of the NF-*κ*B and the mitogen-activated protein kinase (MAPK) that the cell suffers during the initial phase of injury, which leads to the unspecific expression of genes that condition the phenotypical change of the cell in the production of enzymes and the proteins mediated by the TGF*β* and signaling pathways of the SMAD proteins [49, 50] (Figure 3).

## 3. Activation of the Receptors in RAAS by I/R

**Figure 4:**
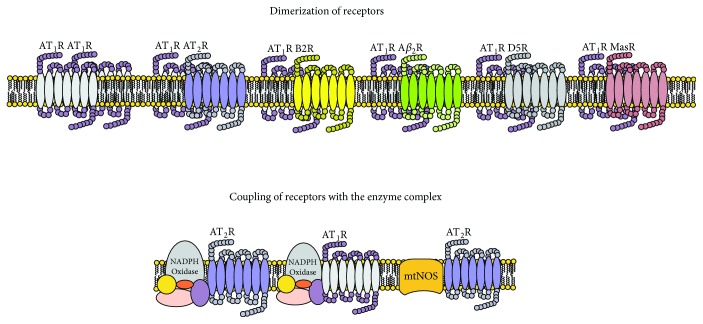
Dimerization and coupling of receptors.

Injury from activation of the RAAS system in the acute phase is characterized by a mechanism of adaptation to the assault, and as the event of I/R is established, the genes involved are overexpressed in maintaining the adaptation, unchaining the substitution of injured tissue with fibrous tissue, changes in the function or phenotype of the cell (transcriptional reprogramming), hypertrophy, cell division, or apoptosis [14, 51]. These changes produce lesions that in the long term diminish the functionality of the affected organs, perpetuating the injury and the chronic activation of the system. The processes are mediated by the activation of receptors belonging to the RAAS among which are the family of Ang receptors (AT_1_R, AT_2_R, AT_4_R, and AT_7_R), the prorenin receptor, the MAS receptor (MASR), and the MET (METR) receptor; the last of which has an activity mediated by the insulin-regulated aminopeptidase receptor (IRAP) [52, 53]. The expression of these receptors is located in multiple cell groups and in the cellular membrane; some of which are found in the cytoplasm in a hydrosoluble form, and others are anchored in the membranes of organelles like in the mitochondria [30, 54]. The family of the ATR, MASR, and METR has the structure typical of the type 7 transmembrane receptors (7TMR) with the extreme N-terminal and three extracellular loops (ECL) and with the extreme C-terminal, three intracellular loops (ICL), and intracellular amphipathic helix VIII, where the conformation of the helices II and III is identical in these receptors. The binding sites to the receptor are the helices II and III of ECL 1, the helices IV and V of ECL 2, and the helices VI and VII of ECL 3 [55]. C-terminal helix VIII shows an effector domain regulated by Ca^++^ and mediated by calmodulin that is necessary for the coupling and activation of the G protein [21]. Activation of the receptor and the uncoupling of the G protein are mediated by the “stretch-induced” process (mechanical stress) by a conformational change in the structure of the TM7R. It can also be activated by allosteric binding mediated by sodium, lipids, and cholesterol, which demonstrates that the receptor, physiologically, can have an active or inactive state without the presence of a specific agonist [46, 56]. These receptors coupled to G proteins have dimerization capabilities in up to 80%. They can present as homodimers (AT_1_R-AT_1_R) or as heterodimers (AT_1_R-B2 bradykinin, AT_1_R-*β*_2_ adrenergic, AT_1_R-D5 dopamine, AT_1_R-Mas, AT_1_R-AT_2_R, and AT_2_R-B2) located in the cellular membrane, physiologically, or in states of stress of the same cell, modulating multiple signaling pathways through direct activation of the receptor or by indirect activation or inactivation of the paired receptor [57] (Figure 4).

**Figure 5:**
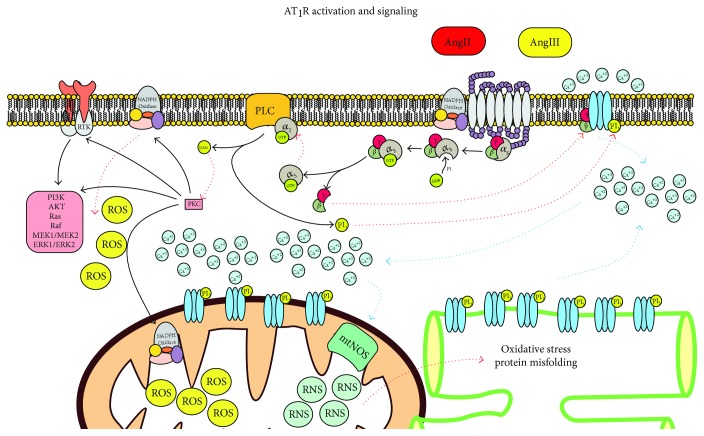
AT_1_R signaling. The activity of the receptor will stimulate oxidative stress and activation of other signaling pathways.

Specifically to the AT_1_R, primarily the G_q/11_ (Ca^++^ mobilizers) proteins can couple secondarily to the proteins G_i/o_, G_12/13_, and G_s_ or the class of Rho, Ras, and Rac [58]. The signaling pathway of the AT_1_R (Figure 5) is also found coupled to other signaling pathways mediated by the receptors of the tyrosine kinase type (RTK), mitogen-activated protein kinase (MAPK), and Janus kinase-signal transducers and activators of transcription (JAK-STAT) [58]. The activation of AT_1_R during I/R injury produces the modulation of ion channels through the activity of the beta-gamma complex of the G protein (G*βγ*) with the production of ROS by activation of the NADPH oxidase enzyme complex, cellular differentiation through activation of the signaling of MAPK, PI3K, and JAK-STAT, and fibrosis through activation of the RTK that promotes differentiation, activation, and proliferation of fibroblasts that will suffer activation of the metalloproteinases coupled with the membrane (ADAM and MMP) [58]. Hypertrophy and a decrease in apoptosis are produced by the activation of the Ras, Raf, MEK1/MEK2, extracellular signal-regulated kinase type 1 and type 2 (ERK1/ERK2), and inflammation through the activation of the NF-*κ*B factor and STAT [59] (Figure 5).

The AT_2_R is 34% similar to the AT_1_R in its amino acid sequence, and the structure is typical of the G protein-coupled receptor (GPCR). However, studies where they have achieved designs of the tridimensional structure (from its crystallized form) demonstrate that the receptor has an intracellular section in the H8 domain that, depending on its position, favors or impedes the coupling of what could be the G protein or the bond with *β*-arrestin for it to be internalized [26]. It has been described that the AT_2_R receptor can dimerize with the bradykinin receptor (B2R) in the cell membrane and with the mtNOS in the mitochondrial matrix, which, on activation, increase the production of NO in the two microenvironments [60, 61]. While the presence of NO in the cytoplasm increases the production of cGMP in the mitochondria, the activation of mtNOS and the increase in NO bring about blockage of complex IV of the mitochondrial respiratory chain and an increase in the RNS like the peroxynitrite (ONOO^−^) [62, 63]. This activity during the early phase of I/R injury will activate the mtNOS to produce changes in the redox signaling that could increase the injury. However, the late phase of injury produces vasodilation and activation of the cGMP signaling involved in the activation of apoptosis and the decrease in vascular proliferation, inhibition of cellular proliferation of the injured tissue, fibrosis, hypertrophy, and regeneration [64, 65]. The dimerization of the AT_2_R-AT_1_R produces direct inhibition of the AT_1_R receptor. Another mechanism of action described for the AT_2_R is the activation of phosphatases (protein phosphatase 2a, MAP kinase phosphatase, and the SH2 domain-containing tyrosine phosphatase-1) that dephosphorylate the signaling pathways activated by the AT_1_R and are activated by the RTKs, MAPK, JAK/SATA, and ERK1/ERK2, which bring the inhibition of cellular proliferation and differentiation, activation of fibroblasts, and activation of the proapoptotic pathways [50]. The activation of AT_2_R also reduces inflammation by inactivation of the signaling pathway mediated by RTKs that activate through phosphorylation of the inhibitor IK*κ*B of the NF-*κ*B, decreasing the concentrations of IL-6 and other proinflammatory cytokines [66].

**Figure 6:**
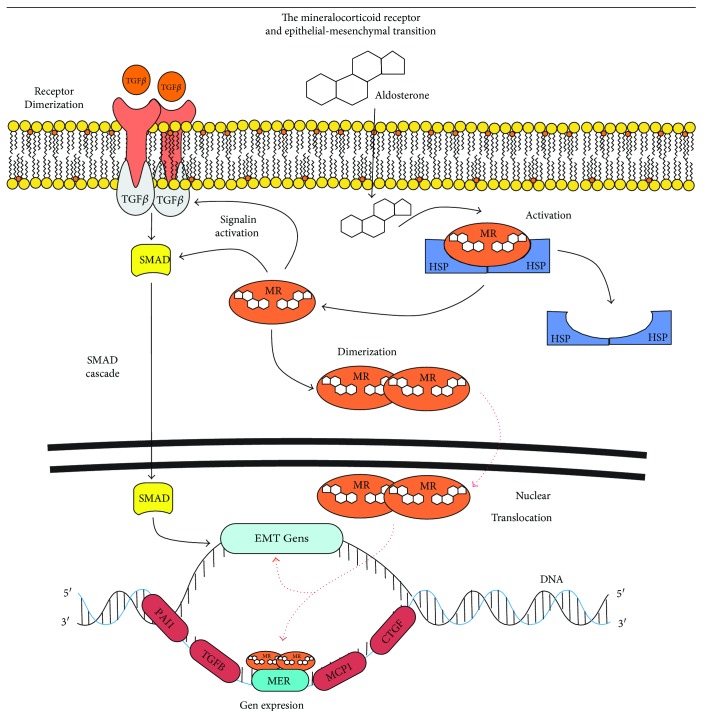
The mineralocorticoid receptor and the epithelial-mesenchymal transition activation.

The mineralocorticoid receptor (MR) is a nuclear receptor that contains 984 amino acids with three functional domains, an extreme N-terminal activation function domain (AF-1), a middle DNA-binding domain (DBD), and a C-terminal ligand-binding domain (LBD) [67]. Activation of the AF-1 and DBD is controlled by hormone binding in the LBD that contains an activation function-2 domain (AF-2) which can be modulated by the activity of hormones or by heat shock proteins (HSP), nuclear translocation, and the recruitment of transcriptional cofactors [68]. The MR is found in the cell cytoplasm and accomplishes functions as a monomer or dimer: it homodimerizes and this gives it the capacity to translocate to the nucleus in order to have contact with the elements of response to the mineralocorticoids (Figure 6). The MR have two levels of action: one in the cytoplasm and the other in the nucleus [69, 70], interacting with multiple signaling pathways with the TGF*β*1, the monocyte chemoattractant protein 1 (MCP1), the connective tissue growth factor (CTGF), and the plasminogen activator inhibitor type 1 (PAI-1). The MR has the ability to activate the NADPH oxidase anchored in the cell membrane, increasing the formation of oxidative stress that leads to the activation of the signaling pathways and the accumulation of extracellular matrix that produces hypertrophy, fibrosis, and alterations in the stability and activity of ion channels [69, 70]. It has been observed that the overactivation of the mineralocorticoids produces alterations in the NO metabolism, decreasing the production of the tetrahydrobiopterin and uncoupling of the NOS [69, 70].

**Figure 7:**
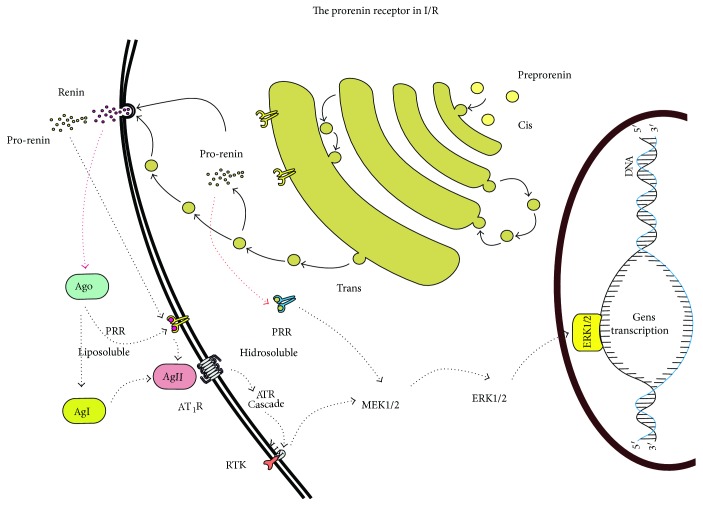
The activation of prorenin receptor during I/R.

The prorenin receptor (Figure 7) has 350 amino acids with only one transmembrane domain, very similar to that of the receptors of the growth factors. In fact, there are no known proteins homologous to the receptor. The receptor has the affinity for renin and prorenin [11] and does not degrade or internalize, is always located on the membrane surface, and has the ability to interact with Ang I, and this process activates the receptor [12]. The activity of the PRR unchains the activity of the signaling pathways of the MAPK and ERK1/ERK2, and the activation of the signaling pathways unchains the overexpression of the TGF*β* receptor, which brings about fibrosis and cellular proliferation. During the phenomenon of I/R, the receptors' expression and activity are increased, which signifies their critical role in perpetuation of the I/R injury in the long term [71].

## 4. Oxidative Stress and RAAS in I/R

Oxidative stress is considered the state of disequilibrium between antioxidant defenses and the excessive production of ROS, among which are the superoxide anion (O^2−^), the hydrogen peroxide (H_2_O_2_), and the hydroxyl radical (OH^**·**^), whose fundamental characteristic is based on exceeding the antioxidant defenses. There are several enzyme systems that contribute to the formation of ROS, including the NADPH oxidase, the xanthine oxidase, and the leakage of mitochondrial electrons of the mitochondrial electron transport chain (METC). The ROS are normally generated as a natural by-product of oxygen metabolism and play an important role in cell signaling. However, when ROS levels augment dramatically under pathologic conditions such as cardiac insufficiency, I/R, and aging, oxidative stress is produced [29]. The NADPH oxidase generates the production of ROS and unchains additional ROS by the mitochondria [55]. The mitochondria are the primary source of ROS production in the cells. Complex I and complex III of the METC are implicated in the generation of oxygen to the O^2−^ anion. Ang II facilitates the uncoupling of the NOS through the increase in the production of the O^2−^ anion, and the uncoupled NOS additionally increases levels of the O^2−^ anion in the vasculature and accelerates endothelial dysfunction. Hence, Ang II is considered a potent activator of the NADPH oxidase. The ROS derived from the NADPH oxidase promote the generation of ROS from the mitochondria [72]. The clinical relevance of Ang II induced by the ROS is unclear, and evidence suggests that inhibition of Ang II decreases the risk of cardiovascular regeneration and inflammation in the I/R process. Various modulation pathways of direct action that Ang II exerts on the ROS have been identified. The link between the oxidative stress and clinical cardiac disturbances is potentially of exceptional importance in the development of diverse therapeutic strategies directed toward the cascades of transduction of the ROS by Ang II, because diverse studies support the critical role of the system in the pathophysiology of damage caused by myocardial ischemia from reperfusion [73]. Therefore, it is considered that the administration of AT_1_ receptor blockers is efficacious in reducing the reperfusion damage caused by myocardial ischemia. Losartan is an AT_1_ receptor blocker that was initially introduced for its hypotensive action, although other studies have demonstrated that, apart from its hemodynamic effects, it could have cardioprotective influences directly affecting the myocardiocytes. Losartan is capable of augmenting the ventricular fibrillation threshold and decreasing the size of an infarct and of augmenting the number of premature cardiac beats, episodes of ventricular tachycardia, and the mortality rate [74]. Nonetheless, losartan reduces the production of ROS on inhibiting the NADPH oxidase enzyme and increasing the production of NO in the endothelial cells on activating the NOS [75].

The sirtuins (SIRT1–SIRT7) are a class of proteins that are characterized by the deacetylation of the histones and a wide range of nonhistone proteins such as enzymes and transcription factors. The sirtuins undertake a fundamental role in the regulation of biological cell activities like metabolism, cell death, cell growth, and the cellular responses to oxidative stress [76]. Sirtuin 3 is a mitochondrial sirtuin that is located completely within the cellular nucleus [77, 78]. Because the mitochondria are entirely involved in the production of energy and metabolism of the heart and because mitochondrial dysfunction is an important factor in the deterioration of ventricular contractions [79], sirtuin 3 regulates the mitochondrial function through deacetylation, the modulation of mitochondrial proteins involved in metabolism, mitochondrial dynamics, and the responses to oxidative stress [80]. Sirtuin 3 protects the heart against oxidative stress from the activation of the family of transcription factors O3a (FoxO3a) that augment the transcription of the gene codifiers for antioxidants like the manganese superoxide dismutase (MnSOD) and the catalase [58, 81]. Vitamin D deficiency is associated directly or indirectly with many traditional cardiovascular risk factors like diabetes mellitus, hypertension, and dyslipidemia [82]. The levels and the effect of vitamin D are fundamental in the modulation of the RAAS system, cardiac contractility, and the proliferation of smooth muscle that leads to hypertrophy of the left ventricle [83]. The greatest source of vitamin D is exposure to sunlight and the endogenous production in the skin, although it can also be obtained, to a lesser degree, from dietary sources. Vitamin D deficiency is considered a problem in different populations, from young to old, and is frequent in patients with cardiac failure [84].

## 5. Mitochondrial Function and Activity of the RAAS in I/R

**Figure 8:**
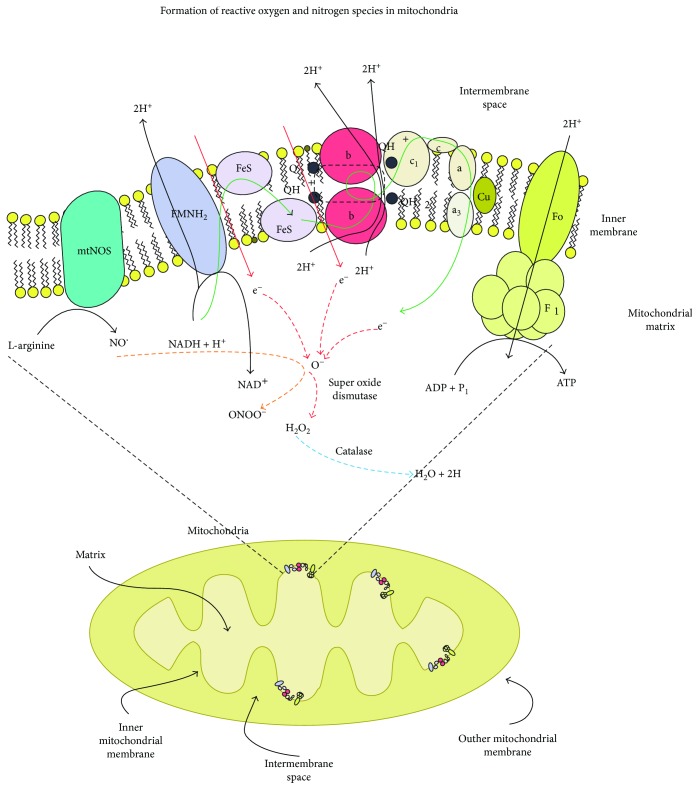
Formation of oxidative stress in mitochondria by I/R.

The mitochondria are energy-producing organelles that carry out diverse, key cellular functions including the regulation of the cytosolic Ca^++^ levels [85] and the oxygen gradient of the tissues [63, 86], the signaling of H_2_O_2_, and the modulation of apoptosis [87] (Figure 8). The mitochondria play a crucial role in the cellular responses to a great variety of stimuli. They receive, integrate, and transmit signals. Mitochondrial damage can lead to functional alterations of the tissues [88]. The mitochondria utilize >90% of the cellular oxygen, while they transform most of it into water through complex IV of the METC. Approximately 1-2% of the oxygen consumed [89] receives electrons directly from complexes I and III to form the O^2−^ anion [90]. Other production sources of mitochondrial ROS (mtROS) include electrons derived from substrates of complex II that can be transported to oxygen and complex I [69], matrix enzymes [70], the external membrane [91], and the uncoupled mitochondrial NOS (mtNOS) [92]. The O^2−^ anion is liberated from the mitochondrial matrix and from the mitochondrial intermembrane space where it is converted into H_2_O_2_ by the MnSOD and the copper-zinc superoxide dismutase (CuZnSOD) [93]. The H_2_O_2_ can be detoxified to water through the action of the mitochondrial enzyme glutathione peroxidase (GPx) or to water and oxygen by the catalase by the cardiac mitochondria. These enzymes belong to a complex, multilevel system of mitochondrial defense, composed of nonenzymatic antioxidant enzymes that participate in the detoxification of ROS [94]. The mitochondria generate other oxidants derived from the NO on producing RNS, which include the ONOO^−^ anion that is formed when the O^2−^ and the NO react. Thus, the mitochondria are relevant formulators of ROS and RNS, which leads to mitochondrial dysfunction with alteration to the generation of energy (ATP) on augmenting the generation of ROS [95].

Ang II stimulates mitochondrial dysfunction in the cardiac smooth muscle, kidney, and vascular cells [96]. Inhibition of Ang II decreases the liberation of mitochondrial ROS, increases the efficiency of the METC, protects mitochondrial structures, and favors the production of ATP. The ACE inhibitors and the AT_1_ receptor blockers reduce mitochondrial dysfunction related to age, attenuate the renal mitochondrial dysfunction induced by hypertension, and protect against cardiac mitochondrial dysfunction from acute ischemia [97]. The mitochondria that malfunction seem to be implicated in the pathogenesis of diverse illnesses. One study showed that the inhibition of the ACE (enalapril) in aged rats impeded the reduction of the number of mitochondria [98]. In studies performed with Ang II marked with 1–125, Ang II was detected in the heart, brain, and smooth muscle mitochondria and nuclei [99]. In the adrenal glomerulosa zone in rats, the renin, AGT, and ACE were detected in the intramitochondrial dense bodies [100]. The immunoreaction of Ang II was observed in the mitochondria of the rat cerebral cortex. Consequently, it is possible to speculate that Ang II can have direct actions on the mitochondria independently of the signaling of the AT_1_ receptor [101]. The renal and cardiac benefits of the ACE inhibitors and AT_1_ receptor blockers of Ang II in patients with arterial hypertension, cardiovascular illness, and diabetes mellitus seem to be partly [102] independent of their effects in reducing arterial pressure [27]. That suggests that the ACE inhibitors and AT_1_ receptor blockers of Ang II can exert direct effects on the tissues in addition to the hemodynamic effects. In this sense, the RAAS inhibitors act effectively against oxidative stress and mitochondrial dysfunction [103]. There are international recommendations for the use of Ang II inhibitors as first-line drugs for kidney protection in diabetics, even in the absence of hypertension [104].

### 5.1. Endoplasmic Reticulum Stress in I/R

The ER is an important intracellular organelle responsible for the synthesis, folding, and trafficking of a wide variety of proteins, hormones, enzymes, receptors, ion channels, and transporters. In normal situations, there is homeostatic equilibrium between the inflow of displayed peptides and the folding capacity in the ER. However, when unfolded or misfolded proteins accumulate, ER stress (ERS) occurs and a specific adaptive response of the cells is triggered to prevent the abnormal accumulation of unfolding proteins. The ERS activates intracellular signal transduction pathways that together are known as the unfolded protein response (UPR) [105]. The UPR is characterized by three phases: (a) an adaptive phase, (b) an alarm phase, and (c) an activation phase of cell death. The adaptation phase is characterized by proteasome degradation of proteins and the subsequent global inhibition of protein transduction leading to the increase in the group of chaperones and protein enzymes such as the X-box 1-binding protein (XBP-1) and the activation of transcription factor 4 (ATF4) [106]. This process limits the ERS by reducing the load of misfolded proteins. The alarm phase consists of the induction of an inflammatory process by activating the N-terminal kinase pathways of NF-*κ*B and c-Jun. If these mechanisms are insufficient to alleviate the misfolded protein load, the ERS activates the immune response against cellular stress and promotes cell death by apoptosis or necrosis [107]. The injury by I/R of transplanted grafts is considered one of the crucial problems that complicate posttransplant care of patients by influencing short- and long-term results. In all organ transplants and in other situations in which I/R occurs, cold preservation and hot reperfusion are temporarily produced; therefore, cell damage occurs in the organ that worsens after the restoration of the oxygen supply [108]. Because of the I/R, the ultrastructural examination of the cells subject to the I/R process reveals abundant amounts of rough and uniform ER. Therefore, it can be considered that the UPR/ERS response contributes to the prevention of the mediation of pathological changes. In this sense, there is increasing evidence that disturbances of ER are new subcellular effectors involved in ischemia [109]. The insult by I/R stimulates the intracellular Ca^++^ overload of the ER lumen, which in turn modulates the mitochondrial vulnerability of Ca^++^ and apoptosis, favoring the release of cytochrome c and the activation of caspases. Once ER homeostasis has been exacerbated, new expanded proteins accumulate and UPR is activated. This occurs during the cold storage of the graft in preservation solutions. UPR/ERS alterations increase after reperfusion, which are determinants for the graft result after transplantation [110]. On the other hand, the RAAS system has a multitude of electrophysiological effects with the capacity to produce cardiac arrhythmia through various mechanisms. An experimental study reported that proarrhythmic effects are suggested on the ion channels of the sarcoplasmic reticulum and of the membrane and that the increase in oxidative stress probably contributes to the higher arrhythmic incidence mediated by the RAAS [111].

### 5.2. Calcium Homeostasis in I/R

There is a large driving electrochemical force that tends to translocate Ca^++^ to cells through two components; the difference in the concentration of Ca^++^ in the extracellular fluids (concentration of extracellular fluids > 10,000 than the intracellular fluids), in addition to the electrical potential through the plasma membranes. The interior of the plasma membrane is 60–90 mV, and for the outside, the value is negative. The electrochemical potential is maintained because the Ca^++^ fluxes associated with signal transduction are small and tightly regulated and because the increase in Ca^++^ triggers the extrusion of Ca^++^ by the exchange of 3Na^+^-Ca^++^ and by a Ca^++^-2H^+^ exchanger driven by ATP. The leakage ratio of the pump maintains Ca^++^ at values of 100–200 nmol/L, although the concentration increases transiently during the activation of the cell [112]. Ca^++^ enters the cell through multiple pathways; additional channels can even be opened under adverse conditions, for example, nonspecific cation channels and others activated by ROS as per H_2_O_2_. However, in the depolarization and accumulation of intracellular Na^+^, the 3Na^+^-Ca^++^ exchanger is able to operate in reverse mode, which causes Ca^++^ to accumulate in the cell [113]. The concentrations of intracellular and extracellular Ca^++^ (normal free) are approximately 0.1 and 1000 *μ*mol/L, respectively. However, the total content of cellular Ca^++^ (excluding extracellular spaces) is approximately 1000 *μ*mol/L (i.e., 1 mmol/L·kg^−1^); it is deduced that >99% of the total cellular Ca^++^ content is found bound to proteins or phospholipids or sequestered in the ER in the calciosomes and mitochondria. Although the source of the increase in Ca^++^ is often due to extracellular Ca^++^, the cell contains enough Ca^++^. The uncontrolled release of Ca^++^ predisposes to cell damage [114]. During ischemia, the extracellular Ca^++^ decreases to values < 10% approximately 0.1 mmol/L, and the extracellular fluid decreases to ~50% of the control. This means that almost all extracellular Ca^++^ is translocated to cells. With an extracellular fluid of 20% tissue volume and an extracellular Ca^++^ of ~1.3 mmol/L, the load of Ca^++^ to which the cells are exposed is ~250 *μ*mol/kg of tissue. Since it is unlikely that much Ca^++^ uptake will occur in the cells, the Ca^++^ load could double that number, which means that the total Ca^++^ content of the cells can increase to ≥150% of the control. Long-term focal ischemia leads to pan necrosis and death of all cell types [115]. Therefore, vascular damage is a prominent feature of ischemic lesions causing a strong inflammatory response [116]. In the 1980s, it was reported that ischemia was accompanied by translocation of Ca^++^ from extracellular fluids to intracellular fluids and that reperfusion restored extracellular Ca^++^ to normal in 15–20 min, without knowing whether changes occurred in the total content of Ca^++^ in tissues [117, 118]. In 1984, results of transient ischemia of the brain < 20 min of duration were reported, which revealed greater incorporation of Ca^++^ in parts of the brain after 24 h of reperfusion [119]. The highest incorporation appeared to occur without an increase in the total cellular Ca^++^ content [114]. Based on these results, another group explored the time course of the changes in the total concentration of tissue Ca^++^ correlating it with microscopic data of cell death and found that the total content of tissue Ca^++^ during reperfusion increased between 24 and 48 h and that the increase in intracellular Ca^++^ apparently preceded the morphological signs of cell death [120].

### 5.3. Apoptosis in I/R

Apoptosis regulates cell turnover in healthy living organisms by eliminating excessive or unhealthy cells. Apoptosis plays a fundamental role in the development of tissues and aging. The apoptotic process consists of two main pathways, the death receptor pathway (extrinsic pathway) and the mitochondrial pathway (intrinsic) [121–124]. The activation of the extrinsic pathway begins through interactions mediated by transmembrane receptors. The death receptors form a subset of the tumor necrosis factor receptor (TNFR) family, which consist of a cytoplasmic domain of approximately 80 amino acids known as the “death domain.” The death domain plays a critical role in the transmission of death signals from the cell surface to the intracellular signaling pathways through the binding of ligands (FasL/FasR) to their corresponding death receptors (TNF-*α*/TNFR1). The receptor-ligand interactions result in the grouping of receptors and the recruitment and oligomerization of adapter proteins (FADD to the Fas receptor). Following the recruitment and oligomerization of FADD, procaspase-8 binds to FADD through the dimerization of its effector death domain resulting in the formation of the death-inducing signaling complex (DISC) and the autocatalytic activation of the caspase-8. The activation of caspase-8 triggers the execution phase of apoptosis [125]. The intrinsic pathway of apoptosis can be initiated by a variety of physical, chemical, and pathophysiological stimuli including ERS, oxidative damage to DNA, metabolic stress, cellular stress caused by ionizing radiation, heat, hypoxia, cytokine deprivation, and chemotherapeutic drugs. These stimuli indicate a complex interaction of Bcl-2 proteins that triggers the phase of apoptosis manifested by the apoptotic pore formation, the loss of mitochondrial transmembrane potential, and the release of cytochrome c and Smac/DIABLO from the intermembrane space to the cytosol. These pathways converge in an execution pathway that involves the activation of caspase-3/7 by caspase-8/9 for the onset of cell death. As a result, the cell is formed into small apoptotic bodies that are swallowed by phagocytosis [126]. The extrinsic and intrinsic pathways are not completely independent of each other, and often the activation of one will induce the other pathway [97, 127, 128]. In the ER, there are damage sensors with the ability to bind to the apoptotic pathways and lead to the activation of caspase-12 [129]. In I/R injury, the ischemia injury is effectively managed with organ reperfusion; however, reperfusion leads to several additional lesions, including activation of the two pathways of apoptosis in the injured organ producing mitochondrial dysfunction, Ca^++^ overload, and overproduction of ROS [130].

## 6. Autophagy: A Process of Cellular Adaptation in I/R

Autophagy is a highly conserved process of bulk protein degradation, responsible for the rotation and clearance of proteins and the elimination of organelles [131]. Autophagy executes an important role in the elimination of misfolded proteins to maintain cellular homeostasis and maintain healthy cells. Autophagy is a process of intracellular lysosome degradation that acts through homeostatic clearance of the organelles and protein aggregates [132]. Autophagy involves the isolation of cellular proteins and organelles, forming autophagosomes (double-membrane structures) that are directed toward the lysosomes [133]. The formation of autophagosomes is dependent on the induction of various genes including the LC3, phosphatidylinositide-3-kinase, Beclin 1, and the Atgs [134]. There are at least three different autophagy paths:

- Macroautophagy (called autophagy) is the nonselective multistep process through which portions of the cytoplasm and/or organelles are isolated in vesicular structures that include mitochondria (mitophagy) known as autophagosomes. The autophagosome fuses with the lysosomes and degrades the autophagosomic contents.
- Microautophagy occurs when the cytosolic load is isolated directly in the lysosome through engulfment of the lysosomal membrane.
- Chaperone-mediated autophagy (CMA) occurs when the misfolded proteins of the cytosol are degraded [135].

An efficient flow through the autophagy pathway is essential for cell survival [136]. The frequent observation of autophagosomes in dying cells has stimulated interest in examining autophagy as a mechanism of cell death (programmed cell death type 2) [137]. The induction of autophagy is critical for survival during the perinatal period of hypoxia and relative starvation [138]. Autophagy is rapidly induced in the myocardium in response to stress from prolonged fasting [139], the excess of pressure [140], and I/R injury. The autophagy induced by stress has been attributed to a healthy role as damaging to the function of myocardiocytes and survival [141]. The activation of autophagy is determined by the speed of autophagosome formation and the rate of autophagosome destruction [136]. It is unclear if the abundance of autophagosomes in dying cells reflects positive regulation of the adaptive autophagy [142], deregulated and excessive autocannibalism, and the deterioration of the flow of autophagy with the reduced elimination of autophagosomes in the activation of programmed cell death [143, 144]. In the heart, autophagy is produced at low levels under normal conditions, but excessive and/or defective autophagy can lead to severe cardiac pathology and finally to cardiac failure [145]. The elevated quantity of vacuoles associated with autophagy that are detected in myocardiocytes in cardiac failure in different human organisms is a fact [146]. The overregulation of autophagy in myocardiocytes together with hypertrophic remodeling after chronic treatment with Ang II has been reported [147]. Ang II contributes to the progression of kidney injury through its hemodynamic effects [148]. The hemodynamic effects are confirmed on diminishing its production and blocking the binding to its receptors [149]. However, apart from its hemodynamic effects, the direct effects of Ang II on the kidney cells are recognized more and more frequently [150]. In the past, the production of Ang II in the kidney has been attributed solely to the specialized cells, yet it has been demonstrated that the majority of kidney cells, including podocytes, mesangial cells, and the epithelial tubular cells, are capable of generating Ang II [151]. It has been suggested that the local concentration of Ang II in the kidney is probably greater than systemic levels of Ang II in blood. Therefore, the direct biological effects of Ang II on kidney cells are perhaps more critical in renal injury [29] since Ang II induces oxidative stress in diverse kidney cells [152]. Also, it has been reported that the oxidative stress induces the process of autophagy. The induction of autophagy is necessary for the elimination of damaged proteins and organelles (oxidized protein aggregates and damaged mitochondria) [153]. The increase in autophagy can cause reduction of the mitochondrial mass by 50%, while at the same time, it reduces the susceptibility of the cells to apoptotic stimuli dependent on the permeability of the external mitochondrial membrane [144]. The exposure to Ang II or the overexpression of the G protein coupled to the AT_1_ receptor induces the excessive production of ROS and unchains autophagy, senescence, and apoptosis in the myocardiocytes and smooth muscle cells. However, if the activity of the RAAS rises, there are benefits with the use of ACE inhibitors or AT_1_ receptor blockers in diabetic patients without cardiovascular complications, although this fact remains controversial [154].

## 7. Therapeutic Targets in the Modulation of the RAAS Activity during I/R

Based on what has been observed, the activation of the signaling pathways for AT_1_R at the onset of the injury is necessary for the cells or injured tissue to have the capacity to survive the ischemic assault. However, the subsequent pathological activation of these receptors during the early phase of I/R injury and the overactivation of the signaling pathways and the genes involved will lead to the epithelial-mesenchymal transition, fibrosis, hypertrophy, and cellular proliferation. For this reason, it is important to understand that blocking the signaling pathways in the ischemic phase will likely not improve the evolution of the initial injury, yet modulation of the signaling pathways in the subsequent phases will favor the limitation of damage and can probably reverse it Table 1.

## 8. Perspectives in RAAS and I/R

The elements of the RAAS are considered potent activators of the NADPH oxidase: the ROS derived from the NADPH oxidase promote the generation of ROS from the mitochondria. Ang II and the other active metabolites facilitate the uncoupling of the NOS, favoring an increase in the production of the O^2−^ anion and of the uncoupled NOS, increasing the levels of the O^2−^ anion in the vasculature, and accelerating endothelial dysfunction. The mitochondria play a critical role in the cellular responses to a great variety of stimuli. The mitochondria receive, integrate, and transmit signals and activate metabolic processes and the processes of adaptation. Mitochondrial damage can lead to dysfunction of the organelle with alterations in the functioning of the tissues. Ang II stimulates mitochondrial dysfunction in the cells of cardiac smooth muscle and kidney and vascular cells, and its inhibition decreases the liberation of mitochondrial ROS, augmenting efficiency of the METC, protecting the mitochondrial structures, and favoring the production of ATP. The local concentration of Ang II in the kidney is, it seems, greater than systemic levels of Ang II. Treatments for the regulation of the RAAS are well known, such as the ARBs, ACEI, renin inhibitors, MR inhibitors, and inhibitors of aldosterone and antioxidant production; yet, despite being able to rely on this number of drugs, the dosage and standardization of a regimen for the management of I/R injury have still not been established. The recommendation would be to start the medications based on an understanding of the physiological processes of the I/R injury and activation of the RAAS. The end result is not to block the response entirely but rather to modulate the exaggerated response of the RAAS when faced with I/R injury.

## Figures and Tables

**Table 1:** Therapeutic targets in the RAAS modulation.

Family	Molecular mechanism	Cell signaling modulation	Proposal therapeutic indication
AT_1_ inhibitors	Direct and selective blockage of the receptor	Survival, apoptosis, autophagy, TME, and fibrosis	After the first 72 h
ACE inhibitors	Blockage of the activity of the ACE	Apoptosis, proliferation, and fibrosis	In the late phase of I/R injury 7–14 days after injury
Renin inhibitors	Direct inhibition	Apoptosis, proliferation, and fibrosis	In the first hours of the injury
Mineralocorticoid receptor antagonists	Direct blockage of the receptor	Survival, apoptosis, autophagy, TME, and fibrosis	In the first hours of the injury
Aldosterone synthase inhibitors	Inhibition in the activity of the enzyme	Survival, apoptosis, autophagy, TME, and fibrosis	In the first hours of the injury
Modulation in the redox signaling and oxidative stress	Oxidative scavengers	Survival, apoptosis, autophagy, TME, and fibrosis	Before the injury and during the whole I/R phenomenon

## References

[1] Murry C. E., Jennings R. B., Reimer K. A. (1986). Preconditioning with ischemia: a delay of lethal cell injury in ischemic myocardium. *Circulation*.

[2] Turer A. T., Hill J. A. (2010). Pathogenesis of myocardial ischemia–reperfusion injury and rationale for therapy. *The American Journal of Cardiology*.

[3] Kalogeris T., Baines C. P., Krenz M., Korthuis R. J. (2012). Cell biology of ischemia/reperfusion injury. *International Review of Cell and Molecular Biology*.

[4] Ruiz-Ortega M., Esteban V., Egido J. (2007). The regulation of the inflammatory response through nuclear factor-*κ*B pathway by angiotensin IV extends the role of the renin angiotensin system in cardiovascular diseases. *Trends in Cardiovascular Medicine*.

[5] Gomez R. A. (2017). Fate of renin cells during development and disease. *Hypertension*.

[6] Brown N. J. (2013). Contribution of aldosterone to cardiovascular and renal inflammation and fibrosis. *Nature Reviews Nephrology*.

[7] Rodríguez-Lara S. Q., Cardona-Muñoz E. G., Ramírez-Lizardo E. J. (2016). Alternative interventions to prevent oxidative damage following ischemia/reperfusion. *Oxidative Medicine and Cellular Longevity*.

[8] Blundell T., Sibanda B. L., Pearl L. (1983). Three-dimensional structure, specificity and catalytic mechanism of renin. *Nature*.

[9] Hobart P. M., Fogliano M., O’Connor B. A., Schaefer I. M., Chirgwin J. M. (1984). Human renin gene: structure and sequence analysis. *Proceedings of the National Academy of Sciences of the United States of America*.

[10] Gomez R. A., Belyea B., Medrano S., Pentz E. S., Sequeira-Lopez M. L. S. (2014). Fate and plasticity of renin precursors in development and disease. *Pediatric Nephrology*.

[11] Yeh P. Y., Yeh K. H., Chuang S. E., Song Y. C., Cheng A. L. (2004). Suppression of MEK/ERK signaling pathway enhances cisplatin-induced NF-*κ*B activation by protein phosphatase 4-mediated NF-*κ*B p65 Thr dephosphorylation. *Journal of Biological Chemistry*.

[12] Azhar M., Runyan R. B., Gard C. (2009). Ligand-specific function of transforming growth factor beta in epithelial-mesenchymal transition in heart development. *Developmental Dynamics*.

[13] Rahimi Z. (2016). The role of renin angiotensin aldosterone system genes in diabetic nephropathy. *Canadian Journal of Diabetes*.

[14] Kurihara T., Ozawa Y., Shinoda K. (2006). Neuroprotective effects of angiotensin II type 1 receptor (AT1R) blocker, telmisartan, via modulating AT1R and AT2R signaling in retinal inflammation. *Investigative Ophthalmology & Visual Science*.

[15] Cook K. M., Hogg P. J. (2013). Post-translational control of protein function by disulfide bond cleavage. *Antioxidants & Redox Signaling*.

[16] Lumbers E. R., Pringle K. G. (2014). Roles of the circulating renin-angiotensin-aldosterone system in human pregnancy. *American Journal of Physiology-Regulatory, Integrative and Comparative Physiology*.

[17] Fukamizu A., Takahashi S., Seo M. S. (1990). Structure and expression of the human angiotensinogen gene. Identification of a unique and highly active promoter. *The Journal of Biological Chemistry*.

[18] Donoghue M., Hsieh F., Baronas E. (2000). A novel angiotensin-converting enzyme–related carboxypeptidase (ACE2) converts angiotensin I to angiotensin 1–9. *Circulation Research*.

[19] Opie L. (2001). Renoprotection by angiotensin-receptor blockers and ACE inhibitors in hypertension. *The Lancet*.

[20] Gohlke P., Weiss S., Jansen A. (2001). AT1 receptor antagonist telmisartan administered peripherally inhibits central responses to angiotensin II in conscious rats. *Journal of Pharmacology and Experimental Therapeutics*.

[21] van Kats J. P., Schalekamp M. A. D. H., Verdouw P. D., Duncker D. J., Danser A. H. J. (2001). Intrarenal angiotensin II: interstitial and cellular levels and site of production. *Kidney International*.

[22] Jackman H. L., Massad M. G., Sekosan M. (2002). Angiotensin 1–9 and 1–7 release in human heart: role of cathepsin A. *Hypertension*.

[23] De Mello W. C. (2004). Angiotensin (1–7) re-establishes impulse conduction in cardiac muscle during ischaemia-reperfusion. The role of the sodium pump. *Journal of the Renin-Angiotensin-Aldosterone System*.

[24] Araki K., Masaki T., Katsuragi I., Tanaka K., Kakuma T., Yoshimatsu H. (2006). Telmisartan prevents obesity and increases the expression of uncoupling protein 1 in diet-induced obese mice. *Hypertension*.

[25] Pachauri P., Garabadu D., Goyal A., Upadhyay P. K. (2017). Angiotensin (1–7) facilitates cardioprotection of ischemic preconditioning on ischemia–reperfusion-challenged rat heart. *Molecular and Cellular Biochemistry*.

[26] Wruck C. J., Funke-Kaiser H., Pufe T. (2005). Regulation of transport of the angiotensin AT2 receptor by a novel membrane-associated Golgi protein. *Arteriosclerosis, Thrombosis, and Vascular Biology*.

[27] Santos R. A. S., Ferreira A. J., Simões e Silva A. C. (2008). Recent advances in the angiotensin-converting enzyme 2–angiotensin (1–7)-Mas axis. *Experimental Physiology*.

[28] Wright J., Yamamoto B., Harding J. (2008). Angiotensin receptor subtype mediated physiologies and behaviors: new discoveries and clinical targets. *Progress in Neurobiology*.

[29] Wen H., Gwathmey J. K., Xie L.-H. (2012). Oxidative stress-mediated effects of angiotensin II in the cardiovascular system. *World Journal of Hypertension*.

[30] Abadir P. M., Foster D. B., Crow M. (2011). Identification and characterization of a functional mitochondrial angiotensin system. *Proceedings of the National Academy of Sciences of the United States of America*.

[31] Bosnyak S., Jones E. S., Christopoulos A., Aguilar M.-I., Thomas W. G., Widdop R. E. (2011). Relative affinity of angiotensin peptides and novel ligands at AT_1_ and AT_2_ receptors. *Clinical Science*.

[32] Brown N. J. (2005). Aldosterone and end-organ damage. *Current Opinion in Nephrology and Hypertension*.

[33] Shimoni Y., Chen K., Emmett T., Kargacin G. (2008). Aldosterone and the autocrine modulation of potassium currents and oxidative stress in the diabetic rat heart. *British Journal of Pharmacology*.

[34] Messaoudi S., Azibani F., Delcayre C., Jaisser F. (2012). Aldosterone, mineralocorticoid receptor, and heart failure. *Molecular and Cellular Endocrinology*.

[35] Briones A. M., Nguyen Dinh Cat A., Callera G. E. (2012). Adipocytes produce aldosterone through calcineurin-dependent signaling pathways: implications in diabetes mellitus–associated obesity and vascular dysfunction. *Hypertension*.

[36] Nolly M. B., Caldiz C. I., Yeves A. M. (2014). The signaling pathway for aldosterone-induced mitochondrial production of superoxide anion in the myocardium. *Journal of Molecular and Cellular Cardiology*.

[37] Maron B. A., Zhang Y.-Y., Handy D. E. (2009). Aldosterone increases oxidant stress to impair guanylyl cyclase activity by cysteinyl thiol oxidation in vascular smooth muscle cells. *The Journal of Biological Chemistry*.

[38] Newfell B. G., Iyer L. K., Mohammad N. N. (2011). Aldosterone regulates vascular gene transcription via oxidative stress–dependent and–independent pathways. *Arteriosclerosis, Thrombosis, and Vascular Biology*.

[39] Shibata S., Nagase M., Yoshida S., Kawachi H., Fujita T. (2007). Podocyte as the target for aldosterone: roles of oxidative stress and Sgk1. *Hypertension*.

[40] Hattangady N. G., Olala L. O., Bollag W. B., Rainey W. E. (2012). Acute and chronic regulation of aldosterone production. *Molecular and Cellular Endocrinology*.

[41] Patni H., Mathew J. T., Luan L., Franki N., Chander P. N., Singhal P. C. (2007). Aldosterone promotes proximal tubular cell apoptosis: role of oxidative stress. *American Journal of Physiology-Renal Physiology*.

[42] Queisser N., Schupp N. (2012). Aldosterone, oxidative stress, and NF-*κ*B activation in hypertension-related cardiovascular and renal diseases. *Free Radical Biology & Medicine*.

[43] Manna P. R., Soh J.-W., Stocco D. M. (2011). The involvement of specific PKC isoenzymes in phorbol ester-mediated regulation of steroidogenic acute regulatory protein expression and steroid synthesis in mouse Leydig cells. *Endocrinology*.

[44] Miller W. L. (2007). Steroidogenic acute regulatory protein (StAR), a novel mitochondrial cholesterol transporter. *Biochimica et Biophysica Acta (BBA) - Molecular and Cell Biology of Lipids*.

[45] Sato R. (2010). Sterol metabolism and SREBP activation. *Archives of Biochemistry and Biophysics*.

[46] Muehlfelder M., Arias-Loza P.-A., Fritzemeier K. H., Pelzer T. (2012). Both estrogen receptor subtypes, ER*α* and ER*β*, prevent aldosterone-induced oxidative stress in VSMC via increased NADPH bioavailability. *Biochemical and Biophysical Research Communications*.

[47] Ding W., Guo H., Xu C., Wang B., Zhang M., Ding F. (2016). Mitochondrial reactive oxygen species-mediated NLRP3 inflammasome activation contributes to aldosterone-induced renal tubular cells injury. *Oncotarget*.

[48] Gross G. J., Auchampach J. A. (2007). Reperfusion injury: does it exist?. *Journal of Molecular and Cellular Cardiology*.

[49] Zhang A., Jia Z., Guo X., Yang T. (2007). Aldosterone induces epithelial-mesenchymal transition via ROS of mitochondrial origin. *American Journal of Physiology-Renal Physiology*.

[50] Zavadil J., Haley J., Kalluri R., Muthuswamy S. K., Thompson E. (2008). Epithelial-mesenchymal transition. *Cancer Research*.

[51] Kalluri R., Weinberg R. A. (2009). The basics of epithelial-mesenchymal transition. *The Journal of Clinical Investigation*.

[52] Zhang H., Unal H., Gati C. (2015). Structure of the angiotensin receptor revealed by serial femtosecond crystallography. *Cell*.

[53] Lu H., Cassis L. A., Vander Kooi C. W., Daugherty A. (2016). Corrigendum: structure and functions of angiotensinogen. *Hypertension Research*.

[54] Wilson B. A., Nautiyal M., Gwathmey T. M., Rose J. C., Chappell M. C. (2016). Evidence for a mitochondrial angiotensin-(1–7) system in the kidney. *American Journal of Physiology-Renal Physiology*.

[55] Zorov D. B., Filburn C. R., Klotz L.-O., Zweier J. L., Sollott S. J. (2000). Reactive oxygen species (ROS-induced) ROS release. *Journal of Experimental Medicine*.

[56] Zhang H., Han G. W., Batyuk A. (2017). Structural basis for selectivity and diversity in angiotensin II receptors. *Nature*.

[57] Ming D., Songyan L., Yawen C. (2017). *trans*-Polydatin protects the mouse heart against ischemia/reperfusion injury *via* inhibition of the renin–angiotensin system (RAS) and Rho kinase (ROCK) activity. *Food & Function*.

[58] Qiu X., Brown K., Hirschey M. D., Verdin E., Chen D. (2010). Calorie restriction reduces oxidative stress by SIRT3-mediated SOD2 activation. *Cell Metabolism*.

[59] Dinh Q. N., Young M. J., Evans M. A., Drummond G. R., Sobey C. G., Chrissobolis S. (2016). Aldosterone-induced oxidative stress and inflammation in the brain are mediated by the endothelial cell mineralocorticoid receptor. *Brain Research*.

[60] Brand M. D., Nicholls D. G. (2011). Assessing mitochondrial dysfunction in cells. *Biochemical Journal*.

[61] Zhao Z., Vinten-Johansen J. (2006). Postconditioning: reduction of reperfusion-induced injury. *Cardiovascular Research*.

[62] Obal D., Dettwiler S., Favoccia C., Scharbatke H., Preckel B., Schlack W. (2005). The influence of mitochondrial KATP-channels in the cardioprotection of preconditioning and postconditioning by sevoflurane in the rat *in vivo*. *Anesthesia & Analgesia*.

[63] Brookes P. S., Salinas E. P., Darley-Usmar K. (2000). Concentration-dependent effects of nitric oxide on mitochondrial permeability transition and cytochrome *c*release. *The Journal of Biological Chemistry*.

[64] Batinic-Haberle I., Tovmasyan A., Roberts E. R. H., Vujaskovic Z., Leong K. W., Spasojevic I. (2014). SOD therapeutics: latest insights into their structure–activity relationships and impact on the cellular redox-based signaling pathways. *Antioxidants & Redox Signaling*.

[65] Kalyanaraman B. (2013). Teaching the basics of redox biology to medical and graduate students: oxidants, antioxidants and disease mechanisms. *Redox Biology*.

[66] Morgan M. J., Liu Z. G. (2011). Crosstalk of reactive oxygen species and NF-*κ*B signaling. *Cell Research*.

[67] Nagase M., Fujita T. (2013). Role of Rac1–mineralocorticoid-receptor signalling in renal and cardiac disease. *Nature Reviews Nephrology*.

[68] Li Y., Suino K., Daugherty J., Xu H. E. (2005). Structural and biochemical mechanisms for the specificity of hormone binding and coactivator assembly by mineralocorticoid receptor. *Molecular Cell*.

[69] Grivennikova V. G., Vinogradov A. D. (2006). Generation of superoxide by the mitochondrial complex I. *Biochimica et Biophysica Acta (BBA)-Bioenergetics*.

[70] Messner K. R., Imlay J. A. (2002). Mechanism of superoxide and hydrogen peroxide formation by fumarate reductase, succinate dehydrogenase, and aspartate oxidase. *The Journal of Biological Chemistry*.

[71] Saris J. J., 't Hoen P. A. C., Garrelds I. M. (2006). Prorenin induces intracellular signaling in cardiomyocytes independently of angiotensin II. *Hypertension*.

[72] Maack C., Böhm M. (2011). Targeting mitochondrial oxidative stress in heart failure. *Journal of the American College of Cardiology*.

[73] Baker K. M., Booz G. W., Dostal D. E. (1992). Cardiac actions of angiotensin II: role of an intracardiac renin-angiotensin system. *Annual Review of Physiology*.

[74] Safari F., Hajizadeh S., Shekarforoush S., Bayat G., Foadoddini M., Khoshbaten A. (2011). Influence of ramiprilat and losartan on ischemia reperfusion injury in rat hearts. *Journal of the Renin-Angiotensin-Aldosterone System*.

[75] Messadi-Laribi E., Griol-Charhbili V., Pizard A. (2007). Tissue kallikrein is involved in the cardioprotective effect of AT1-receptor blockade in acute myocardial ischemia. *The Journal of Pharmacology and Experimental Therapeutics*.

[76] Lavu S., Boss O., Elliott P. J., Lambert P. D. (2008). Sirtuins—novel therapeutic targets to treat age-associated diseases. *Nature Reviews Drug Discovery*.

[77] Pillai V. B., Sundaresan N. R., Jeevanandam V., Gupta M. P. (2010). Mitochondrial SIRT3 and heart disease. *Cardiovascular Research*.

[78] Samant S. A., Zhang H. J., Hong Z. (2014). SIRT3 deacetylates and activates OPA1 to regulate mitochondrial dynamics during stress. *Molecular and Cellular Biology*.

[79] Tompkins A. J., Burwell L. S., Digerness S. B., Zaragoza C., Holman W. L., Brookes P. S. (2006). Mitochondrial dysfunction in cardiac ischemia–reperfusion injury: ROS from complex I, without inhibition. *Biochimica et Biophysica Acta (BBA) - Molecular Basis of Disease*.

[80] Newman J. C., He W., Verdin E. (2012). Mitochondrial protein acylation and intermediary metabolism: regulation by sirtuins and implications for metabolic disease. *The Journal of Biological Chemistry*.

[81] Sundaresan N. R., Gupta M., Kim G., Rajamohan S. B., Isbatan A., Gupta M. P. (2009). Sirt3 blocks the cardiac hypertrophic response by augmenting Foxo3a-dependent antioxidant defense mechanisms in mice. *The Journal of Clinical Investigation*.

[82] Lee J. H., O’Keefe J. H., Bell D., Hensrud D. D., Holick M. F. (2008). Vitamin D deficiency: an important, common, and easily treatable cardiovascular risk factor?. *Journal of the American College of Cardiology*.

[83] Zittermann A. (2006). Vitamin D and disease prevention with special reference to cardiovascular disease. *Progress in Biophysics and Molecular Biology*.

[84] Lee J. H., Jarreau T., Prasad A., Lavie C., O’Keefe J., Ventura H. (2011). Nutritional assessment in heart failure patients. *Congestive Heart Failure*.

[85] Duszyński J., Kozieł R., Brutkowski W., Szczepanowska J., Zabłocki K. (2006). The regulatory role of mitochondria in capacitative calcium entry. *Biochimica et Biophysica Acta (BBA) - Bioenergetics*.

[86] Brookes P., Darley-Usmar V. M. (2002). Hypothesis: the mitochondrial NO• signaling pathway, and the transduction of nitrosative to oxidative cell signals: an alternative function for cytochrome *C* oxidase. *Free Radical Biology & Medicine*.

[87] Thomas D. D., Liu X., Kantrow S. P., Lancaster J. R. (2001). The biological lifetime of nitric oxide: implications for the perivascular dynamics of NO and O_2_. *Proceedings of the National Academy of Sciences of the United States of America*.

[88] Goldenthal M. J., Marín-García J. (2004). Mitochondrial signaling pathways: a receiver/integrator organelle. *Molecular and Cellular Biochemistry*.

[89] Chance B., Sies H., Boveris A. (1979). Hydroperoxide metabolism in mammalian organs. *Physiological Reviews*.

[90] Turrens J. F. (2003). Mitochondrial formation of reactive oxygen species. *The Journal of Physiology*.

[91] Cadenas E., Davies K. J. A. (2000). Mitochondrial free radical generation, oxidative stress, and aging. *Free Radical Biology & Medicine*.

[92] Brodsky S. V., Gao S., Li H., Goligorsky M. S. (2002). Hyperglycemic switch from mitochondrial nitric oxide to superoxide production in endothelial cells. *American Journal of Physiology-Heart and Circulatory Physiology*.

[93] Muller F. L., Liu Y., Van Remmen H. (2004). Complex III releases superoxide to both sides of the inner mitochondrial membrane. *The Journal of Biological Chemistry*.

[94] Andreyev A. Y., Kushnareva Y. E., Starkov A. A. (2005). Mitochondrial metabolism of reactive oxygen species. *Biochemistry*.

[95] Shigenaga M. K., Hagen T. M., Ames B. N. (1994). Oxidative damage and mitochondrial decay in aging. *Proceedings of the National Academy of Sciences of the United States of America*.

[96] de Cavanagh E. M. V., Piotrkowski B., Basso N. (2003). Enalapril and losartan attenuate mitochondrial dysfunction in aged rats. *The FASEB Journal*.

[97] Kaufmann S. H., Hengartner M. O. (2001). Programmed cell death: alive and well in the new millennium. *Trends in Cell Biology*.

[98] Molitch M., DeFronzo R., Franz M. (2004). Nephropathy in diabetes. *Diabetes Care*.

[99] Sirett N. E., McLean A. S., Bray J. J., Hubbard J. I. (1977). Distribution of angiotensin II receptors in rat brain. *Brain Research*.

[100] Peters J., Kranzlin B., Schaeffer S. (1996). Presence of renin within intramitochondrial dense bodies of the rat adrenal cortex. *American Journal of Physiology-Endocrinology and Metabolism*.

[101] Erdmann B., Fuxe K., Ganten D. (1996). Subcellular localization of angiotensin II immunoreactivity in the rat cerebellar cortex. *Hypertension*.

[102] Bosch J., Lonn E., Pogue J. (2005). Long-term effects of ramipril on cardiovascular events and on diabetes: results of the HOPE study extension. *Circulation*.

[103] Münzel T., Keaney J. F. (2001). Are ACE inhibitors a “magic bullet” against oxidative stress?. *Circulation*.

[104] Katyare S. S., Satav J. G. (2005). Effect of streptozotocin-induced diabetes on oxidative energy metabolism in rat kidney mitochondria. A comparative study of early and late effects. *Diabetes, Obesity and Metabolism*.

[105] Diaz-Villanueva J. F., Diaz-Molina R., Garcia-Gonzalez V. (2015). Protein folding and mechanisms of proteostasis. *International Journal of Molecular Sciences*.

[106] Oslowski C. M., Urano F. (2011). Measuring ER stress and the unfolded protein response using mammalian tissue culture system. *Methods in Enzymology*.

[107] Xu C., Bailly-Maitre B., Reed J. C. (2005). Endoplasmic reticulum stress: cell life and death decisions. *The Journal of Clinical Investigation*.

[108] Van Riel W. G., van Golen R. F., Reiniers M. J., Heger M., van Gulik T. M. (2016). How much ischemia can the liver tolerate during resection?. *HepatoBiliary Surgery and Nutrition*.

[109] Zhou H., Zhu J., Yue S. (2016). The dichotomy of endoplasmic reticulum stress response in liver ischemia-reperfusion injury. *Transplantation*.

[110] Montalvo-Jave E. E., Escalante-Tattersfield T., Ortega-Salgado J. A., Pina E., Geller D. A. (2008). Factors in the pathophysiology of the liver ischemia–reperfusion injury. *Journal of Surgical Research*.

[111] Iravanian S., Dudley S. C. (2008). The renin-angiotensin-aldosterone system (RAAS) and cardiac arrhythmias. *Heart Rhythm*.

[112] Blaustein M. P. (1988). Calcium transport and buffering in neurons. *Trends in Neurosciences*.

[113] Gorter J. A., Petrozzino J. J., Aronica E. M. (1997). Global ischemia induces downregulation of glur2 mRNA and increases AMPA receptor-mediated Ca^2+^ influx in hippocampal CA1 neurons of gerbil. *The Journal of Neuroscience*.

[114] Paschen W. (1996). Disturbances of calcium homeostasis within the endoplasmic reticulum may contribute to the development of ischemic-cell damage. *Medical Hypotheses*.

[115] Petito C. K., Pulsinelli W. A., Jacobson G., Plum F. (1982). Edema and vascular permeability in ischemia: comparison between ischemic neuronal damage and infarction. *Journal of Neuropathology & Experimental Neurology*.

[116] Hallenbeck J. M. (1996). Inflammatory reactions at the blood-endothelial interface in acute stroke. *Advances in Neurology*.

[117] Harris R. J., Symon L., Branston N. M., Bayhan M. (1981). Changes in extracellular calcium activity in cerebral ischaemia. *Journal of Cerebral Blood Flow & Metabolism*.

[118] Siemkowicz E., Hansen A. J. (1981). Brain extracellular ion composition and EEG activity following 10 minutes ischemia in normo- and hyperglycemic rats. *Stroke*.

[119] Dienel G. A. (1984). Regional accumulation of calcium in postischemic rat brain. *Journal of Neurochemistry*.

[120] Deshpande J. K., Siesjö B. K., Wieloch T. (1987). Calcium accumulation and neuronal damage in the rat hippocampus following cerebral ischemia. *Journal of Cerebral Blood Flow & Metabolism*.

[121] Czabotar P. E., Lessene G., Strasser A., Adams J. M. (2014). Control of apoptosis by the BCL-2 protein family: implications for physiology and therapy. *Nature Reviews Molecular Cell Biology*.

[122] Elmore S. (2007). Apoptosis: a review of programmed cell death. *Toxicologic Pathology*.

[123] Ichim G., Tait S. W. G. (2016). A fate worse than death: apoptosis as an oncogenic process. *Nature Reviews Cancer*.

[124] Tait S. W. G., Green D. R. (2010). Mitochondria and cell death: outer membrane permeabilization and beyond. *Nature Reviews Molecular Cell Biology*.

[125] Brown J. M., Attardi L. D. (2005). The role of apoptosis in cancer development and treatment response. *Nature Reviews Cancer*.

[126] Fulda S., Debatin K.-M. (2006). Extrinsic versus intrinsic apoptosis pathways in anticancer chemotherapy. *Oncogene*.

[127] Chen H. C., Kanai M., Inoue-Yamauchi A. (2015). An interconnected hierarchical model of cell death regulation by the BCL-2 family. *Nature Cell Biology*.

[128] Wong R. S. Y. (2011). Apoptosis in cancer: from pathogenesis to treatment. *Journal of Experimental & Clinical Cancer Research*.

[129] Gong L., Tang Y., An R., Lin M., Chen L., Du J. (2017). RTN1-C mediates cerebral ischemia/reperfusion injury via ER stress and mitochondria-associated apoptosis pathways. *Cell Death & Disease*.

[130] Li H., Wang Y., Wei C. (2015). Mediation of exogenous hydrogen sulfide in recovery of ischemic post-conditioning-induced cardioprotection via down-regulating oxidative stress and up-regulating PI3K/Akt/GSK-3*β* pathway in isolated aging rat hearts. *Cell & Bioscience*.

[131] Lee Y., Lee H.-Y., Gustafsson Å. B. (2012). Regulation of autophagy by metabolic and stress signaling pathways in the heart. *Journal of Cardiovascular Pharmacology*.

[132] Yang Z., Klionsky D. J. (2010). Mammalian autophagy: core molecular machinery and signaling regulation. *Current Opinion in Cell Biology*.

[133] Kuma A., Hatano M., Matsui M. (2004). The role of autophagy during the early neonatal starvation period. *Nature*.

[134] Xie Z., Klionsky D. J. (2007). Autophagosome formation: core machinery and adaptations. *Nature Cell Biology*.

[135] Orogo A. M., Gustafsson Å. B. (2015). Therapeutic targeting of autophagy: potential and concerns in treating cardiovascular disease. *Circulation Research*.

[136] Iwai-Kanai E., Yuan H., Huang C. (2008). A method to measure cardiac autophagic flux in vivo. *Autophagy*.

[137] Galluzzi L., Morselli E., Vicencio J. M. (2008). Life, death and burial: multifaceted impact of autophagy. *Biochemical Society Transactions*.

[138] Kroemer G., Levine B. (2008). Autophagic cell death: the story of a misnomer. *Nature Reviews Molecular Cell Biology*.

[139] Zhu H., Tannous P., Johnstone J. L. (2007). Cardiac autophagy is a maladaptive response to hemodynamic stress. *Journal of Clinical Investigation*.

[140] Nakai A., Yamaguchi O., Takeda T. (2007). The role of autophagy in cardiomyocytes in the basal state and in response to hemodynamic stress. *Nature Medicine*.

[141] Matsui Y., Takagi H., Qu X. (2007). Distinct roles of autophagy in the heart during ischemia and reperfusion: roles of AMP-activated protein kinase and beclin 1 in mediating autophagy. *Circulation Research*.

[142] Kroemer G., Galluzzi L., Vandenabeele P. (2009). Classification of cell death: recommendations of the Nomenclature Committee on Cell Death 2009. *Cell Death & Differentiation*.

[143] Klionsky D. J. (2004). Cell biology: regulated self-cannibalism. *Nature*.

[144] Rubinsztein D. C., Gestwicki J. E., Murphy L. O., Klionsky D. J. (2007). Potential therapeutic applications of autophagy. *Nature Reviews Drug Discovery*.

[145] Rothermel B. A., Hill J. A. (2007). Myocyte autophagy in heart disease: friend or foe?. *Autophagy*.

[146] Yan L., Vatner D. E., Kim S.-J. (2005). Autophagy in chronically ischemic myocardium. *Proceedings of the National Academy of Sciences of the United States of America*.

[147] Porrello E. R., D’Amore A., Curl C. L. (2009). Angiotensin II type 2 receptor antagonizes angiotensin II type 1 receptor–mediated cardiomyocyte autophagy. *Hypertension*.

[148] Matsusaka T., Hymes J., Ichikawa I. (1996). Angiotensin in progressive renal diseases: theory and practice. *Journal of the American Society of Nephrology*.

[149] Taal M. W., Brenner B. M. (2000). Renoprotective benefits of RAS inhibition: from ACEI to angiotensin II antagonists. *Kidney International*.

[150] Bhaskaran M., Reddy K., Radhakrishanan N., Franki N., Ding G., Singhal P. C. (2003). Angiotensin II induces apoptosis in renal proximal tubular cells. *American Journal of Physiology-Renal Physiology*.

[151] Singh R., Singh A. K., Alavi N., Leehey D. J. (2003). Mechanism of increased angiotensin II levels in glomerular mesangial cells cultured in high glucose. *Journal of the American Society of Nephrology*.

[152] Lodha S., Dani D., Mehta R. (2002). Angiotensin II-induced mesangial cell apoptosis: role of oxidative stress. *Molecular Medicine*.

[153] Mizushima N. (2007). Autophagy: process and function. *Genes & Development*.

[154] Xiong S., Salazar G., Patrushev N. (2013). Peroxisome proliferator-activated receptor *γ* coactivator-1*α* is a central negative regulator of vascular senescence. *Arteriosclerosis, Thrombosis, and Vascular Biology*.

